# Using the Electronic Health Record to Facilitate Drug Allergy Delabeling

**DOI:** 10.1007/s11882-025-01229-2

**Published:** 2025-10-25

**Authors:** Matthew J. Molloy, Adam P. Yan, Averi E. Wilson, Jonathan Beus, Lauren M. Hess

**Affiliations:** 1 Divisions of Hospital Medicine and Biomedical Informatics, Department of Pediatrics, University of Cincinnati College of Medicine and Cincinnati Children’s Hospital Medical Center, Cincinnati, OH USA; 2 Division of Hematology/Oncology, The Hospital for Sick Children, Toronto, ON Canada; 3 Clinical Informatics Center, Division of Pediatric Hospital Medicine, Department of Pediatric, University of Texas Southwestern, Dallas, TX USA; 4 Department of Information Systems and Technology, Children’s Healthcare of Atlanta and Division of Hospital Medicine, Department of Pediatrics, Emory University, Atlanta, GA USA; 5 Division of Pediatric Hospital Medicine, Department of Pediatrics, Baylor College of Medicine, Texas children’s Hospital, Houston, TX USA; 6 Division of Hospital Medicine, Cincinnati Children’s Hospital Medical Center, 3333 Burnet Avenue, MLC 3024, Cincinnati, OH 45229 USA

**Keywords:** Allergy delabeling, Electronic health record, Clinical informatics, Clinical decision support

## Abstract

**Purpose of Review:**

There is a growing number of allergy delabeling programs across diverse clinical specialties and care settings. The electronic health record (EHR) can be leveraged to facilitate allergy delabeling. The purpose of this review is to describe EHR tools that have been used in allergy delabeling programs. We also provide recommendations for organizations considering EHR-based allergy delabeling workflows that incorporate clinical informatics best practices.

**Recent Findings:**

Recent literature describes several EHR tools used in delabeling. These tools can be organized around the steps of the allergy delabeling workflow: 1. Identify eligible patients, 2. Risk stratify, 3. Evaluation and testing, 4. Documentation of outcome, 5. Delabeling, and 6. Allergy label reconciliation.

**Summary:**

Standardized EHR tools across the allergy delabeling workflow can lead to successful delabeling and support of diverse stakeholders. Partnering with EHR vendors presents an opportunity to make these tools readily available and improve allergy documentation.

## Introduction

The first decade of the 21 st century saw rapid expansion and uptake of electronic health record (EHR) systems in the United States [1]. In the EHR, drug allergy labels are commonly listed in an allergy section or activity of the patient chart and are most often populated by a patient’s physician, advanced practice provider, nurse, or medical assistant [2, 3]. This allergy section typically allows for the entering of discrete data on the drug or substance the patient is allergic to, reaction details, and reaction severity [4]. The inclusion of allergies and adverse drug reactions in the EHR enhances patient safety, with most mature EHRs able to alert prescribers to potential drug-allergy interactions.

Allergy and adverse drug reaction documentation in the EHR has some notable shortcomings. Details about the reaction and reaction severity are optional in most EHRs and therefore often omitted, resulting in unreliable and difficult-to-interpret information. Studies have identified that 30–50% of allergy labels in the EHR are lacking needed information to risk stratify patients with drug allergy labels [2, 5, 6]. Information about allergy, adverse drug reaction, and drug tolerance can also be found in other areas of the EHR that do not interface with the allergy section, including laboratory results (e.g., antibody testing), flowsheets, and unstructured documentation (e.g., notes), leading to discrepancies that need to be reconciled by the healthcare team [4]. Additionally, most EHRs do not allow for nuanced documentation of adverse reactions, with most documented “allergies” not representing true immune-mediated reactions (e.g., pruritic with opioids or diarrhea after an antibiotic) [7–9]. 

Drug allergy labels are widespread and influence clinical decision making. Upwards of 35% of patients have a drug allergy label listed in their EHR, with one study finding that 25% of patients have 3 or more drug allergy labels [5, 10]. Antibiotics represent the most common drug allergy label, with upwards of 12% of patients having a penicillin allergy label [10, 11]. These labels are often applied early in life, with one multicenter study finding that children were labeled at a median age of 1.3 years [11]. With up to 20% of pediatric ambulatory encounters leading to a prescription for an antibiotic, these antibiotic allergy labels can have a large impact on prescribing patterns [12]. 

The high prevalence of these antibiotic allergy labels is particularly concerning given that many do not represent true IgE-mediated allergies. Overdocumentation of allergies can lead to patients receiving suboptimal therapy, unnecessary exposure to broad spectrum antibiotics, increased risk of infection from drug resistant organisms, adverse drug events, longer lengths of stay, and higher costs [13–15]. This overdocumentation of allergies can also lead to downstream impacts on clinician alert burden. Allergy labels can generate multiple interruptive alerts per encounter and studies have found increasing trends in clinicians overriding allergy alerts, with over 80% of alerts overridden [16, 17]. Concerningly, over 70% of immune-mediated and potential life-threatening interaction alerts were also overridden [16]. 

Despite the widespread frequency of penicillin allergy labels, more than 95% of patients with a penicillin allergy label can receive penicillins without having an adverse reaction [11]. Allergy delabeling is the process of evaluating a patient’s reported allergy, and removing the allergy label from their chart if they tolerate the exposure. Allergy delabeling can occur through direct oral challenge or even through history alone. Skin testing can serve a role as an adjunctive evaluation tool. Numerous studies have demonstrated the effectiveness and safety of these approaches to delabel patients and allow for subsequent safe administration of antibiotics, improving desired prescribing practices and lowering cost [17–20]. Overall, these evaluations accurately identify 9 of 10 patients who can be delabeled and are recommended to be proactively offered to healthy patients in advance of antibiotic need [21]. Allergists and other clinicians are increasingly turning to the EHR and other technologies to assist with the allergy delabeling processes.

In this article, we present a brief review of recent literature on how the EHR can be leveraged to facilitate allergy label evaluation and the allergy delabeling processes, with a focus on penicillin allergy labels as a model which can be applied to other drugs. We also provide recommendations for organizations considering EHR-based allergy delabeling workflows that incorporate clinical informatics best practices.

## Allergy Delabeling and the EHR

**Fig. 1:**
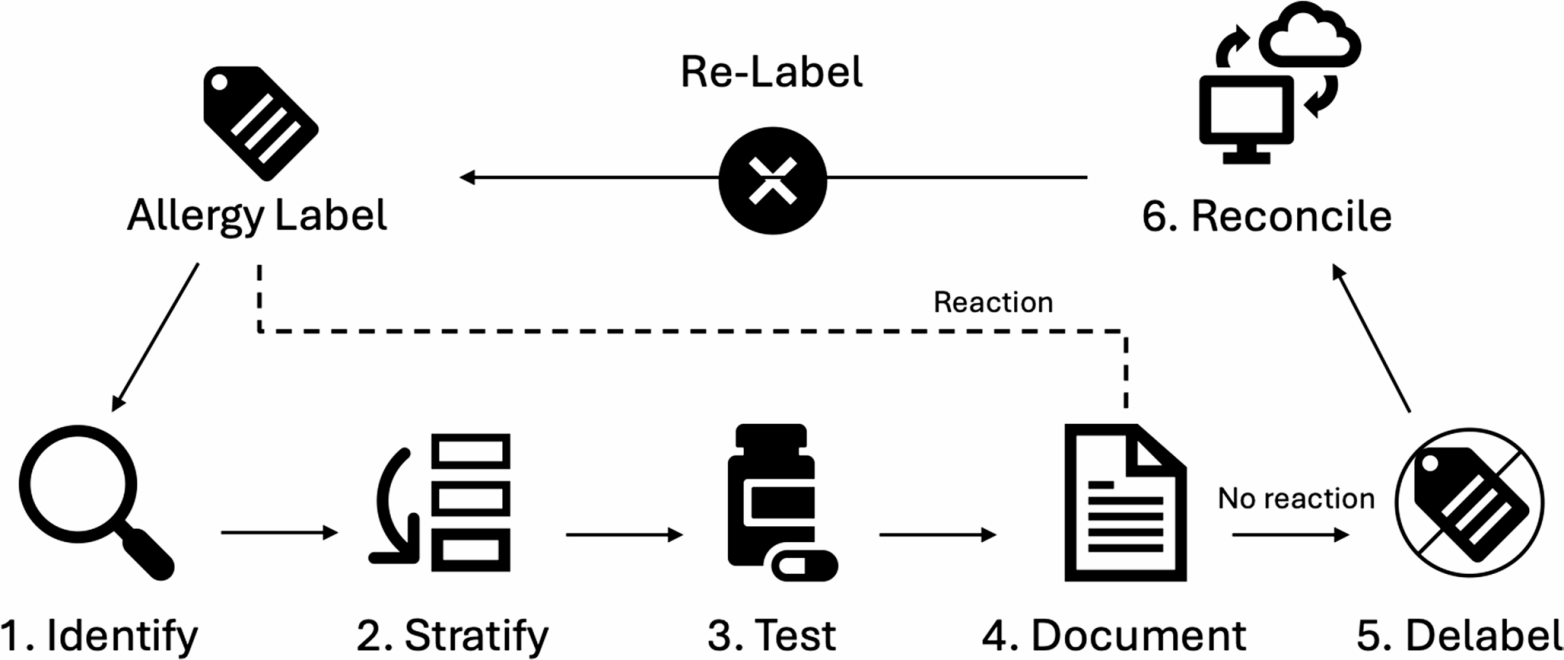
Allergy delabeling worklowf

The expansion of the allergy delabeling process to various practice settings (e.g., emergency department, inpatient units) and non-allergy medical specialties presents an opportunity to leverage EHR tools to provide clinical decision support (CDS) – technology that delivers patient-specific information and recommendations to aid clinical decision-making [22]. The EHR can support the delabeling workflow at several points in the process (Figure 1), with specific EHR tools designed to support each step (Table 1).

**Table 1:** Allergy delabeling workflow EHR tools

Allergy Delabeling Workflow Step	EHR Tool Examples
1. Identify	- Allergy/adverse reaction documentation tools: allergy section/activity, note documentation templates - Patient-level flags or notifications - Patient list columns - Reports - Dashboards
2. Stratify	- Standardized risk stratification tools: questionnaires, flowsheets, other structure documentation tools - Notifications or alerts to prompt stratification - Note documentation templates - Mobile applications - Telehealth
3. Test	- Notifications or alerts to prompt allergy evaluation - Automated referrals - Order-based tools such as order sets
4. Document	- Note documentation templates - Patient education templates - Provider-to-provider communication templates - Structure documentation tools such as flowsheets
5. Delabel	- Notifications or alerts to prompt delabeling - Reports to audit delabeling after successful allergy evaluation
6. Reconcile	- Allergy/adverse reaction documentation tools: documentation in resolved label - Notifications or alerts if a resolved label is readded - Patient education templates - Provider-to-provider communication templates

## Identify

To accurately identify eligible patients and evaluate for delabeling, consistent allergy label documentation is helpful. As discussed previously, the documentation of allergies is often incomplete in the EHR. Yet, with concerted quality improvement work, the completeness of this discrete, non-free text documentation (e.g., reaction type, reaction severity) can be improved to trigger downstream CDS. One multidisciplinary team convened a local workgroup to identify high-risk allergy labels for remediation. They successfully remediated >130,000 labels, using a rule-based automated process for >50,000 of these [23]. Another group introduced a standard section to their history and physical note template asking clinicians to verify type, reaction, and severity of each allergy label, with pharmacists ensuring complete documentation. The process improved the completeness of allergy labels from 20% to 45% over 12 months [24]. 

Delabeling workflows outside of an allergy office generally require EHR tools to help identify eligible patients (i.e., patients with a relevant allergy label). In addition to review of the allergy section or activity, allergy labels and associated details can be gleaned through allergy reconciliation tools, review of the medication administration record, and/or through general chart search functions. Various EHR tools exist to bring additional visibility to a patient’s allergy labels at other points in the clinical workflow. To enhance identification at the patient level, notifications can be configured during the check-in and registration process to flag an eligible patient [25]. Eligible patients might also be identified when the need arises for a specific medication to be used in treatment, generating a flag or alert.

At the clinical or inpatient unit level, columns can be configured to display allergies on a patient list to allow quick review of an entire group of patients [24, 26]. On a hospital or program level, reports and dashboards can be developed to rapidly curate data on a large number of patient to identify eligible patients, and have been successfully incorporated into delabeling programs [24, 26]. 

### Stratify: Allergy History and Risk Stratification

After identification of a patient with a documented allergy who is potentially eligible for delabeling, the next step is to risk stratify their allergy, a process the EHR can support by integrating necessary documentation. Several risk stratification tools and algorithms have been developed and implemented with success, including the Antibiotics Allergy Assessment Tool (AAAT) and PEN-FAST tool for penicillin allergy [17, 27–32]. The specific questions and data fields required to apply these stratification algorithms can be incorporated into the EHR as flowsheets or structured documentation, enabling clinical decision support that informs the provider about the patient’s risk level. Automated prompts or alerts have been used to prompt nursing to complete a patient questionnaire that facilitates risk stratification [33, 34]. Structured documentation templates have also been used by pharmacists to risk stratify patients [24]. 

Notably, several studies have developed and evaluated the use of mobile applications to provide clinical decision support around allergy delabeling [35–37]. These tools can be easily accessed by clinicians and are not specific to a local instance of an EHR. Some of these mobile application tools are explicitly patient facing, such as the Penicillin Allergy Decision and Mobile Empowerment tool [38], and are completed directly by the patient or family.

Most health systems offer telehealth services, which are often integrated into the EHR. In recent years, telehealth has been employed to facilitate evaluation for allergy delabeling, often as a way to risk stratify patients and determine next steps for testing. The use of telehealth has been shown to both improve access to allergy specialists and to increase the number of patients delabeled per month [39–41]. 

### Test

The results of the allergy history and risk stratification drive recommendations for next steps to address the allergy label, which may include delabeling by history alone, oral challenge, referral to an allergist, skin prick testing, or maintenance of the allergy label. Risk stratification results in the EHR can trigger additional clinical decision support. One group developed an ambulatory physician-facing interruptive alert suggesting referral to an allergist for testing following risk stratification [25]. At the same institution, an automated outpatient referral is placed at the time of hospital discharge for risk stratified patients who did not receive inpatient allergy testing [33]. 

Order-based EHR tools can assure standardization and reduce the cognitive burden of performing allergy testing, especially for non-allergy clinicians. Several delabeling programs have utilized order sets and order-based protocols for allergy testing [33, 42]. These tools can be implemented across practice settings and lower the barrier to carrying out testing by providing easily accessible structure for clinicians who perform the allergy evaluation infrequently. These order sets can include oral challenge drug dosing, rescue medications, nursing orders, lab orders, and/or referral orders, when appropriate.

### Document

Documentation of the outcome of allergy testing is an important component of the delabeling process. Documentation templates can facilitate standardization of this documentation and have been used successfully in delabeling programs [24, 33]. Standardized documentation tools can be used both in clinical notes to document results of allergy testing and in the allergy label itself to document date and outcome of testing. Templates like these can help share results with primary care providers, such as in discharge summaries or form letters, and can also be used for printed materials for patients and families.

### Delabel

The goal of delabeling processes is to remove allergy labels from those patients are deemed low risk for or do not demonstrate an immune-mediated reaction during testing or oral challenge. The EHR facilitates this removal by allowing users to discontinue an allergy in a patient’s chart. Unfortunately, this critical step may be inadvertently bypassed if there is no remind in place, as demonstrated by a study that found that penicillin allergy labels remained in place for 28% of patients who passed an oral challenge [43]. In contrast, one team developed an interruptive alert that appeared to clinicians 24 h following an allergy test if the allergy label had not been updated or removed. They found that this alert improved the percentage of updated/removed labels from 51% to 67% updated [44]. In another study, researchers used natural language processing (NLP), a form of artificial intelligence that enables computers to understand and interpret human language, to identify that 7% of patients with successful oral challenge did not have their allergy label removed [45]. 

### Reconcile

Allergy labels in the EHR can be “sticky” due to the difficulty with removing reference to the allergy label completely. Labels can be inappropriately readded by clinicians after chart review, following patient report, or through reconciliation of data from other health systems through health information exchanges (HIEs). While the ability to reconcile patient information like allergies and medications across health systems through interoperability is an incredibly useful and important aspect of modern health information technology, it can make the enduring removal of labels challenging. Even following allergy label removal, clinicians can be presented with the option to reconcile and re-add the same allergy label that gets “pulled in” to the EHR through an HIE. Studies have found that between 6.6 and 7.5% of successfully delabeled patients can be inappropriately re-labeled [4, 46]. EHR tools can be employed to attempt to prevent this relabeling. Hampton et al. developed an alert that appears to future users if they attempt to readd an allergy label that has been deleted [24]. This workflow reinforces the importance of documentation in the allergy label to help prevent future relabeling. A thorough discussion with the patient about why they no longer need to report their allergy and communication of the outcome with primary care providers may also help facilitate persistent removal of the allergy label.

## Recommendations

The EHR can be leveraged throughout the allergy delabeling workflow. Here we present recommendations based on clinical informatics best practice and recent literature presented above for how organizations can operationalize these tools to drive successful delabeling programs.

### Engage Stakeholders

A successful allergy delabeling program implemented at scale requires a multidisciplinary team of stakeholders, which may include allergists, infectious disease specialists, antimicrobial stewardship champions, primary care physicians, emergency medicine physicians, hospitalists, surgeons, nursing, pharmacy, hospital management, clinical informaticists, and patients and caregivers [47]. Though this multidisciplinary group will work closely together and act as champions in their local settings, one of them should serve as the owner of the delabeling program. A lack of ownership has been identified as a major barrier to delabeling initiatives [48]. The owner should be an expert in medication allergies, which may include an allergy specialist, infectious disease specialist, or pharmacist. These experts provide important support to other clinicians and to patients, who may be less willing to accept allergy testing without the involvement of a specialist [49]. The owner and assembled subject matter experts (SME) are incredibly important for the design and implementation of successful CDS tools. In particular, SMEs can ensure that EHR tools are incorporated into clinical workflows, targeting decision support to the right users at the right time in the workflow.

Partnership with both clinical informatics and information technology (IT) is required to build, implement, and support the necessary EHR tools. Currently, most major EHR vendors do not offer out-of-the-box tools to support allergy delabeling and EHR tools such as documentation templates, alerts, and flowsheets need to be built locally. IT support may also be needed to develop new telehealth visit types. Clinical informaticists can bridge the communication between clinical SMEs and IT as well as contribute their expertise in the design and evaluation of CDS. We recommend engaging these teams early in the planning stages of building EHR tools to support a delabeling program.

### Apply Clinical Decision Support Principles

Clinical informatics best practice for the design and implementation of effective CDS includes adhering to the “5 Rights” of CDS – presenting the right information to the right person through the right channel at the right time and in the right format [22, 50]. Critical to the success of any CDS, therefore, is understanding clinical workflows and incorporating CDS tools into these workflows. While many allergy delabeling programs utilize information technology, the majority have not fully integrated the delabeling workflow into the EHR. Patient identification and risk stratification processes (steps 1 and 2 above) often occur outside of the EHR. We recommend that these tools be built into the EHR to facilitate easy access, allow clinical workflow integration, and drive response to risk stratification. For example, the result of a risk stratification flowsheet might trigger an icon in the patient’s chart or an alert to a pharmacist – all made possible by these data points being captured discretely in the EHR.

Several delabeling programs have incorporated interruptive EHR alerts in the delabeling workflow [24, 25, 33, 34, 44]. Interruptive alerts can be a valuable and effective EHR tool if designed well, but these alerts also contribute to clinician alert burden and are often dismissed and disregarded. For interruptive alert build, we recommend consulting a clinical informaticist to ensure adherence to the 5 Rights of CDS. As an example, an alert may be designed to alert clinicians about a patient with an amoxicillin allergy label who has received amoxicillin during their hospitalization. An ideal alert might fire to an ordering provider 12 h after receipt of amoxicillin or when the discharge order is signed, limiting the alert to the right person at the right time. The alert itself should inform the provider why it is appearing and provide a link to the allergy activity to facilitate delabeling, and possibly guidance on what information to enter in the allergy activity.

### Leverage Standardized Tools

When possible, validated and standardized delabeling tools should be built into the EHR as discrete data elements (e.g., flowsheets). For penicillin allergy, this might include the AAAT or PEN-FAST tools [17, 27–32]. Integrating standardized tools into the EHR has several benefits, including improved documentation, standard allergy testing, the ability to use the data to drive other CDS in the EHR, and the facilitation of measurement and reporting of outcomes related to a delabeling program. Implementing standard, validated tools can also facilitate better intraorganizational spread to other care settings and even interorganizational spread [37, 51]. Multiple organizations using the same EHR tools can also allow for multicenter data analysis and research. Lastly, if standardized tools lead to success with one medication (e.g., penicillin), the same build framework can be used to facilitate delabeling of other medications.

### Change Management

The best designed EHR tools will not work if people don’t use them. Implementing a successful delabeling program that involves non-allergist clinicians will require change management and the addressing of potential barriers. For clinicians, these barriers include knowledge about allergy labels, beliefs about potential consequences, and lack of resources, including time [48]. These barriers might be overcome with targeted education and assurance about resources, particularly when questions arise or follow-up is needed. Change management principles, like defining the problem, creating a sense of urgency, and communicating a clear vision, can also help. In non-allergy clinical settings, clinicians also note legitimate competing clinical concerns and demands [52]. This further highlights the importance of workflow integration and incorporating tools into the EHR to decrease barriers to engaging with allergy delabeling processes.

Change management activities will also need to involve patients and families. Patients may hold inaccurate beliefs about allergy labels and the risks associated with future exposure, even following successful allergy testing and delabeling. For example, one study found that 1 year after successful delabeling, 14% of delabeled patients continued to reject penicillin prescriptions [53]. Other studies have also found incomplete willingness to take the medication in the future following delabeling [54]. Patient and family educational materials and communication, which can be facilitated through EHR patient portals, might help to overcome these barriers.

### Monitor Outcomes

Following implementation of a delabeling program, it is important to monitor outcomes. These might include process measures, outcomes measures, and balancing measures. Examples of process measures include how many PEN-FAST assessments performed and how often an order set is used. An example of an outcome measure is the number of patients delabeled. An example of a balancing measure is the number of interruptive alerts that fire in the EHR, a measure of alert burden. The EHR can help to facilitate automated monitoring of these measures to streamline data collection and review. Integrated EHR tools produce data that can be queried for analysis of included in EHR-based reports. Blumenthal et al. also describes validation of an EHR penicillin allergy delabeling prevalence measure with excellent sensitivity and specificity [55]. We recommend selecting metrics and creating a data management plan before embarking on an allergy delabeling program to ensure adequate ability to monitor success. An eye toward future data needs will also be helpful during the build phase of EHR tools.

### Partner with Informatics and EHR Vendors

Lastly, organizations and specialists have an opportunity to partner with clinical informatics professionals and EHR vendors (e.g., Epic Systems, Oracle Health) to work toward development of improve allergy-related EHR tools. As discussed, standardized, validated delabeling tools such as risk stratification flowsheets can be incorporated into EHR vendor build to be offered as out-of-the-box solutions for their customers. This lowers the barrier to every individual organization needing to build EHR tools to facilitate delabeling and promotes both the use of validated tools and the standardized exchange of data.

There is also a significant opportunity to develop improvements to the allergy section/activity of EHRs to improve documentation, prevent over-labeling of allergies, and enhance delabeling efforts. Many groups have called for improvements to this section of the EHR [4, 7–9], noting significant shortcomings of the current state, which lumps all avoidances, potential adverse reactions, and adverse reactions from all agents (e.g., medications, food, chemical, etc.) into one section, leading to an incomplete ability to capture and communicate information with needed complexity.

Workgroups from the American Academy of Allergy, Asthma, and Immunology (AAAAI) have advocated for changing the name of the Allergy section/module to “Adverse Reactions” or “Alerts” [8, 9]. As clinical informaticists who work regularly with a multitude of “alert” types in the EHR, such as sepsis alerts and billing deficiency alerts, we would advocate for “Adverse Reactions” or “Prescribing Considerations” since we believe “Alerts” might lead to confusion. Renaming the section should be paired with development to reconfigure and improve functionality of the section. Ideally, this section would allow for discrete data collection with needed granularity of the agent type (drug, food, chemical etc.), the name of the agent, the date noted, reaction type, severity, and reason for avoidance. In the context of delabeling, we would advocate for fields that capture allergy testing and date of delabeling. During development, the complexity of the problem must be balanced with practical consideration for use, which is why it is critical to involve diverse stakeholders including allergy specialists, general practitioners, and informaticists.

Finally, there is an opportunity to develop improved EHR tools to prevent the reacquisition of removed allergy labels. In combination with the discrete fields to capture important delabeling information noted above, alerts that appear to clinicians if they attempt to re-add a removed allergy label should become standard practice. Development should also occur to improve the interoperability and reconciliation of removed allergy labels across health systems. Developing tools that ease communication with a patient’s primary medical home and pharmacy would go a long way to protect against inappropriate relabeling.

## Conclusion

The EHR offers several CDS tools that can be implemented throughout the allergy delabeling workflow. These tools can improve documentation, increase standardization, and lower the barrier to engaging non-allergy clinicians in the delabeling process. Engaging stakeholders, adhering to CDS best practice, and incorporating change management are critical to success. In light of growing evidence of success in this area, allergists and their allies have an opportunity to partner with clinical informaticists and EHR vendors to make global improvements to allergy workflows in the EHR.

## Data Availability

No datasets were generated or analysed during the current study.

## Key References

- White AA, Ramsey A, Guyer A, Israelsen RB, Khan F, Kaplan B, et al. AAAAI Position Statement on Changing Electronic Health Record Allergy Documentation to “Alerts” to Lead to Easily Understood, Actionable Labels. J Allergy Clin Immunol Pract. 2024;12 [12]:3237-41. **AAAAI Position Statement describing recommended enhancements to the allergy section/activity in EHRs. The authors recommend changing the name of the allergy section to “alerts” and propose functionality to documenting allergies/”alerts” withen an emphasis on meaningful management plans.**
- Powell N, Stephens J, Kohl D, Owens R, Ahmed S, Musicha C, et al. The effectiveness of interventions that support penicillin allergy assessment and delabeling of adult and pediatric patients by nonallergy specialists: a systematic review and meta-analysis. Int J Infect Dis. 2023;129:152 − 61. **Recent meta-analysis of literature through January 2022 evaluating the effectiveness and safety of penicillin allergy delabeling by non-allergy clinicians. Pooled studies represent over 5000 delabeled patients**,** with high effictiveness and safety.**
- Powell N, Elkhalifa S, Guyer A, Garcez T, Sandoe J, Zhou L. Addressing the Challenges of Penicillin Allergy Delabeling With Electronic Health Records and Mobile Applications. J Allergy Clin Immunol Pract. 2023;11 [2]:414 − 21. **Excellent review article about EHR and mobile application tools that can be used to faciliate allergy delabeling. Describes proposed improvements to drug-allergy alerts.**
- Guyer AC, Macy E, White AA, Kuruvilla ME, Robison RG, Kumar S, et al. Allergy Electronic Health Record Documentation: A 2022 Work Group Report of the AAAAI Adverse Reactions to Drugs, Biologicals, and Latex Committee. J Allergy Clin Immunol Pract. 2022;10 [11]:2854-67. **AAAAI work group report describing current shortcomings and proposed changes to the allergy section/activity in EHRs. The authors provide guidance on the definition of specific adverse drug reaction type**,** reconciliation of confirm reactions**,** and removal of erroneous labels.**

## Declarations

### Purpose of Review

There is a growing number of allergy delabeling programs across diverse clinical specialties and care settings. The electronic health record (EHR) can be leveraged to facilitate allergy delabeling. The purpose of this review is to describe EHR tools that have been used in allergy delabeling programs. We also provide recommendations for organizations considering EHR-based allergy delabeling workflows that incorporate clinical informatics best practices.
